# Pain treatment for nursing home residents differs according to cognitive state – a cross-sectional study

**DOI:** 10.1186/s12877-016-0295-1

**Published:** 2016-06-17

**Authors:** Ulrike Bauer, Stefan Pitzer, Maria Magdalena Schreier, Jürgen Osterbrink, Reinhard Alzner, Bernhard Iglseder

**Affiliations:** Institute of Nursing Science and Practice, Paracelsus Medical University Salzburg, Strubergasse 21, A-5020 Salzburg, Austria; Department of Geriatric Medicine, Christian Doppler Klinik, Paracelsus Medical University, Ignaz-Harrer-Straße 79, A-5020 Salzburg, Austria

**Keywords:** Pain, Analgesic treatment, Nursing home residents, Cognitive impairment, Assessment

## Abstract

**Background:**

Communication skills are known to decrease with advancing cognitive impairment. Analgesic treatment in long-term care may be deficient due to the residents’ impaired ability to communicate their pain and needs. Undertreated pain frequently leads to rising BPSD in residents with cognitive impairment, resulting in a treatment with antipsychotics. Aim of this study was the analysis of differences in assessment and pharmacological treatment of pain in nursing home residents relative to their cognitive state and ability to articulate pain.

**Methods:**

Data stems from the baseline of a non-experimental pre-post-study in 12 Austrian nursing homes. Residents’ pain prevalence in relation to pain assessment and cognitive decline was assessed, data on medical diagnoses and prescriptions were retrieved from the nursing homes’ documentation (*n* = 425). Residents were first divided into two groups: Residents with MMSE ≥ 18 were selected into group CUS (cognitively unimpaired/slightly impaired), residents with MMSE ≤ 17 were selected into group CI (cognitively moderately to severely impaired). CI residents were then sub-grouped according to their ability to communicate pain via the Verbal Rating Scale (VRS) (i.e. group CI-V, group CI-NV). Pain behavior of CI residents was assessed with a modified German version of PAINAD. Group differences were tested with ANOVA and H-test, 95 % confidence intervals were calculated and associations were tested with log-binomial regression.

**Results:**

Pain prevalence in CI residents irrespective of their ability to communicate pain was 80 % and exceeded the CUS group prevalence significantly by 14 %. CI residents had significantly less analgesic prescriptions. Furthermore, CI residents have a significantly higher risk of getting no analgesics when in pain than CUS residents (CI-V: RR =2.6, CI-NV: RR =3.4). Use of antipsychotics was high in all groups (49 – 65 %) with more prescriptions in the cognitively impaired group.

**Conclusion:**

Results point toward an underuse of pain medication in cognitively impaired residents, especially those unable to communicate pain verbally. The implementation of standardized pain assessments adapted to the cognitive abilities of residents may foster the recognition of pain, warrant optimized pain management, reduce inadequate medication and consequently raise the chance of equally effective pain treatment regardless of cognitive state.

## Background

Pain is common in older people [1, 2], particularly in nursing home (NH) residents [3, 4] and those with cognitive impairment [5, 6]. Old age exposes individuals to different types of pain, most commonly related to musculoskeletal, gastrointestinal, neurological and cardiac conditions, genitourinary infections, injuries, as well as pressure ulcers in bed ridden individuals, with particularly high pain prevalence rates in those suffering from cognitive impairment [6, 7]. One recently published investigation based on health insurance claims data reported no difference in the diagnoses indicating pain between participants with incident cognitive impairment and cognitively fit controls [8].

Since cognitive impairment is common in many nursing home residents [9, 10], assessment and management of pain is particularly demanding for physicians and nurses due to ambiguity in communication leading to the reasonable assumption that pain in persons with cognitive decline is both under-diagnosed and under-treated [11–13]. In addition, use of analgesics has been reported with higher quality of life in individuals with cognitive impairment [14]. There is evidence that persons with advanced cognitive decline either receive pain treatment, notably opioids, less frequently or in lower insufficient doses as compared to their cognitively fit counterparts [12, 15–19], whereas only few studies have reported a possible overuse of analgesics, particularly paracetamol, in patients with cognitive impairment [20]. On the other hand, there is lack of information to which extent the severity of cognitive decline affects the use of other kind of medication, i.e. antipsychotics, since pain is not only a frequent cause of behavioral and psychological symptoms in dementia (BPSD) [7, 21], but may also, among others, stem from the under-treatment of pain [7, 22]. Pain-induced disruptions run the risk of being misinterpreted as BPSD, provoking inappropriate prescription of psychotropic drugs (i.e. antipsychotics) which, in turn, have been associated with compromised cognition, falls and fractures and increased risk of death [23]. As mentioned above, there is broad consensus within the literature that the challenge of accurately identifying pain in cognitively impaired individuals is the paramount cause of sub-optimal management of pain [24]. Hence in 2009, the American Geriatric Society recommended a comprehensive, disease-specific assessment to establish adequate pain management on an individual level [25]. While individuals with mild to moderate cognitive impairment are often able to report pain either verbally or by use of rating scales [3, 26], these options are not applicable for those with advanced cognitive impairment when the ability to communicate is severely impaired. Thus, self-reported pain may not always be reliable in people with advanced cognitive impairment and pain should be indirectly delineated by raters using a validated observational instrument [27]. Various numerical and visual scales are available for self-reported experience of pain, all of them lacking soundness in persons with cognitive impairment due to their subjection on memory, abstract thinking and speech comprehension [28].

Primary aim of our study was the comparison of assessment and pharmacological treatment of pain in Austrian NH residents in relation to their cognitive state based on self-report and observational assessment of prevalence and intensity of pain, generating the following research questions:

- Are there any differences in the prevalence of pain relating to cognitive state and mode of assessment? Are there differences in diagnoses indicating pain relating to cognitive state?
- Are there any differences in pharmacological pain treatment relating to cognitive state? Are cognitively impaired residents at higher risk of experiencing pain without analgesic medication than their cognitively better performing counterparts?

Since BPSD may be a consequence of undertreated pain, we additionally asked:

- Are cognitively impaired residents at a higher risk to receive antipsychotics?

## Methods

The presented cross-sectional data were collected as part of a baseline investigation of a non-experimental pre-post study with semi-standardized interventions for optimizing pain management in nursing homes (NH) in Austria (OSiA = **O**ptimiertes **S**chmerzmanagement **i**n **A**ltenpflegeheimen, German for: Optimized pain management in nursing homes). Residents’ pain prevalence in relation to pain assessment and cognitive state was assessed and data on medical diagnoses and prescriptions were retrieved from the nursing homes’ documentation (*n* = 425). The study was approved by the ethical committee of Salzburg (415-E/1412/4-2011 v. 07.10.2011). A written consent was obtained from the nursing home residents or from their legal representatives.

### Institutions and study participants

The study was conducted in 12 facilities of one private nursing home company in Austria, which were selected from a total of 29 nursing homes by one-stage cluster sampling. Baseline data were collected in 2011/12. The selected nursing homes are located in seven of the nine Austrian federal states. Participants were recruited by trained study-coordinators. For inclusion and exclusion criteria see Table 1. Potential participants were anonymized via code allocation.

**Table 1:** Inclusion and exclusion criteria

Inclusion and exclusion criteria	
Inclusion criteria	Exclusion criteria
- Age ≥ 60	- Short-term care (up to 6 weeks) and day care
- Living permanently (>3 months) in the NH	- Congenital permanent mental disabilities
- All levels of cognitive impairment	- Insufficient German language skills and/or aphasia
- All levels of physical impairment	- Acute illness and life-threatening situations
- Written consent from resident or his/her legal representative	

### Classification according to cognitive state

A validated German version of the Mini Mental Status Examination (MMSE) [29] as suggested by Kaiser et al. [30] was engaged to classify residents into groups according to their cognitive abilities. Residents with MMSE ≥ 18 were selected into **group CUS** (cognitively unimpaired/slightly impaired), residents with MMSE 17 and lower were selected into **group CI** (cognitively moderately – severely impaired) [31]. CI residents were then sub-grouped dependent on their ability to communicate pain verbally via the Verbal Rating Scale (VRS) [32, 33]: Residents who were able to communicate pain verbally via the VRS were termed **group CI-V** (verbally communicating), residents not able to communicate by VRS were termed **group CI-NV** (not verbally communicating).

### Measurement tools

Data collection was conducted by trained research assistants with experience in geriatric care. Prevalence and severity of pain of CUS were investigated by a standardized questionnaire. Raters used tablets with online versions of the questionnaire [computer assisted personal interview (CAPI)]. The questionnaire included a 5-item Verbal Rating Scale VRS (no – mild – moderate – strong – unbearable pain) [32, 33]. Whenever possible, VRS was also used for CI residents. Pain was assessed at rest and during mobilization (see PAINAD-Gm). Maximum pain was defined as the residents’ maximum pain rating, both at rest or during mobilization.

A modified German version of the Pain Assessment in Advanced Dementia Scale (PAINAD-Gm) [34–36] was engaged for observational assessment of pain behavior of all CI residents. In accordance with [37], PAINAID-Gm was assessed by means of standardized movements (lifting arms, lifting legs, rolling over in bed or getting up from a chair). A cut-off of two or more points out of ten was used to identify residents probably experiencing pain [38]. For group CI-V, maximum pain was recorded as the residents’ maximum pain score either on the VRS or the PAINAD-Gm.

Data on medical diagnoses, prescriptions and level of care were recorded from the nursing homes’ documentation. Diagnoses were categorized according to organ systems with diagnoses most likely associated with pain (according to expert opinion) being labelled ‘Red Diagnoses’ (Table 2).

Medicines prescribed to the residents were classified on the basis of the Anatomical Therapeutic Chemical (ATC) classification system recommended by the WHO [39] additionally distinguishing between prescriptions scheduled permanently (PP) and medications prescribed ‘as needed’ (PRN).

Since the risk of not receiving any analgesics was assumed to be highest in residents who neither had PP analgesics nor PRN analgesics prescribed, analyses regarding the risk of having no analgesic medication though in pain were based on a corresponding subsample.

### Statistical analysis

Sample characteristics are presented as descriptive statistics. Differences between cognition-groups were tested with ANOVA for quantitative variables and with H-test for dichotomous variables. Post-hoc analyses were conducted via *t*-test and Dunn’s test. 95 % confidence intervals and their fluctuation range for group differences were calculated. Associations were tested with log-binomial regression and calculated as risk ratios (RR). Type-1 error (2-sided) was set to 5 % a priori.

## Results

Characteristics of residents are summarized in Table 2. The gender distribution was equal between the three groups. CI-NV residents were significantly older than the others. CI-V and CI-NV residents had a higher classification in the level of care than CUS residents. There were no differences in the length of stay in the NH. CUS residents had a significantly higher number of medical diagnoses than their CI-counterparts, but fewer neurological diagnoses (i.e. dementia). Orthopedic diagnoses were significantly higher in group CUS than in group CI-V. Group CI-NV had significantly less prescriptions scheduled permanently (PP) than the other two groups.

**Table 2:** Characteristics of participants - descriptive statistics

Characteristics of participants				
Groups	All	CUS^b^	CI-V^c^	CI-NV^d^
Sample size (n (%))	425 (100 %)	243 (57.2 %)	116 (27.3 %)	66 (15.5 %)
Sex female (n (%))	315 (74.1 %)	172 (70.8 %)	88 (75.9 %)	55 (83.3 %)
Age (Mean ± SD)	83.6 ± 8.8	82.5 ± 9.4	84.4 ± 7.8	86.1 ± 7.5*
Level of care (Median of range 1–7)	5	4*	5	5
Length of stay in months (Mean ± SD)	29.7 ± 23.9	27.8 ± 20.8	31.2 ± 27.8	34.5 ± 26.3
Number of diseases (Mean ± SD)	7,4 ± 4.3	8,0 ± 4.7*	6.8 ± 4.0	6.5 ± 3.2
Disease categories (n (%))				
Cardiology	305 (73.3 %)	179 (76.2 %)	83 (72.2 %)	43 (65.2 %)
Neurology	295 (70.9 %)	145 (61.7 %)*	91 (79.1 %)	59 (89.4 %)
Endocrinology	228 (54.8 %)	133 (56.6 %)	64 (55.7 %)	31 (47.0 %)
Orthopedics	204 (49.0 %)	128 (54.5 %)*	46 (40.0 %)	30 (45.5 %)
Subgroups of ‘Red Diagnoses’ (n(%)^a^)				
Injuries and trauma sequelae	108 (49.1 %)	67 (48.6 %)	27 (54.0 %)	14 (43.8 %)
Arthrosis	74 (33.6 %)	50 (36.2 %)	13 (26.0 %)	11 (34.4 %)
Disorders of spine and extremities	67 (30.5 %)	49 (35.5 %)	11 (22.0 %)	7 (21.9 %)
Neuropathic	22 (10.0 %)	15 (10.9 %)	4 (8.0 %)	3 (9.4 %)
Number of prescriptions scheduled permanently (PP) (Mean ± SD)	9.0 ± 3.9	9.7 ± 3.9	8.8 ± 3.9	6.8 ± 3.2*

^a^Percentages are based on residents with at least one ‘Red Diagnosis’; significant test results are marked with * (*p* < 0.05)

^b^ cognitively unimpaired/slightly impaired, ^c^ cognitively moderately to severely impaired, verbally communicating, ^d^ cognitively moderately to severely impaired, not verbally communicating

### Prevalence of pain, mode of assessment and diagnoses indicating pain

**Fig. 1:**
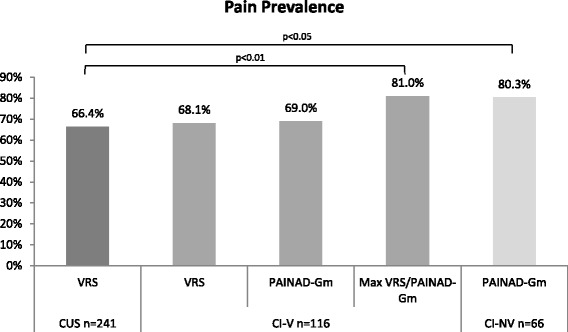
Pain prevalence related to resident group and measurement tool. Notes: VRS low to unbearable pain, PAINAID-Gm cut-off 2, sample CUS had 2 missing data; CUS residents were assessed with the VRS, in CI-V residents VRS and PAINAD-Gm were engaged, CI-NV residents were assessed by PAINAID-Gm

66.4 % of CUS residents presented pain of different intensity at rest or during mobilization. 68.1 % of CI-V residents reported pain by the VRS, whereas the PAINAD-Gm indicated pain in 69.0 %. Counting the maximum pain rating from self-report or proxy assessment generates the best possible sensitivity when screening residents for pain, indicating pain in 81.0 % of group CI-V. In the group CI-NV the pain prevalence was 80.3 % (Fig. 1 and Table 3). Our results demonstrate significant differences in pain prevalence between CUS residents and both groups of CI residents (Table 3).

**Table 3:** Pain prevalence and ‘red diagnoses’ – descriptive statistics and group differences

Pain prevalence and ‘Red Diagnoses’							
	Descriptive statistics				Group differences		
Groups	All	CUS^a^	CI-V^b^	CI-NV^c^	CUS vs. CI-V	CUS vs. CI-NV	CI-V vs. CI-NV
	%	%	%	%	Diff % (±FR)	Diff % (±FR)	Diff % (±FR)
Pain prevalence	72.6	66.4	81.0	80.3	14.6 (±9.2) **	13.9 (±11.3) *	−0.7 (±12.0) n.s.
At least 1 ‘Red Diagnosis’	52.9	58.7	43.5	48.5	−15.2 (±9.2) **	−10.2 (±13.6) n.s.	5.0 (±5.0) n.s.

**p < 0.01, *p < 0.05, *n.s.* not significant, *Diff* Group difference, *FR* Fluctuation range of 95 % confidence interval

^a^ cognitively unimpaired/slightly impaired, ^b^ cognitively moderately to severely impaired, verbally communicating, ^c^ cognitively moderately to severely impaired, not verbally communicating

58.7 % of CUS residents, 43.5 % of CI-V and 48.5 % of CI-NV residents exhibited at least one ‘Red Diagnosis’, displaying a significant difference between CUS and CI-V (Table 3).

### Pharmacological pain treatment

Analgesics were the most commonly used medications in the investigated nursing homes. CUS residents were more likely to have analgesic prescriptions than the CI residents (sum of analgesics PP and PRN). 87.0 % of group CUS got at least one analgesic PP and/or PRN, while this applied to 78.3 % of group CI-V and 76.6 % of group CI-NV. In all groups, more analgesics were prescribed PRN (64.1 – 72.3 %) than PP (36.0 – 58.0 %). In group CI-NV, fewer analgesics were administered PP (36.0 %) than in group CUS (58.0 %), whilst no such significant difference existed between group CI-V and CI-NV (Table 4).

**Table 4:** Prescribed analgesics – descriptive statistics and group differences

Prescribed analgesics							
	Descriptive statistics				Group differences		
Groups	All	CUS^a^	CI-V^b^	CI-NV^c^	CUS vs. CI-V	CUS vs. CI-NV	CI-V vs. CI-NV
					Diff % (±FR)	Diff % (±FR)	Diff % (±FR)
Sum of analgesics (PP and PRN) (Mean ± SD)	2.11 ± 1.64	2.32 ± 1.63	1.90 ± 1.62	1.66 ± 1.61	0.4 (±0.35) *	0.7 (±0.45) **	0.3 (±0.5) n.s.
At least one analgesic (PP and PRN) (%)	83.0	87.0	78.3	76.6	−8.7 (±8.7) *	−10,4 (±11.2) *	−1.7 (±12.8) n.s.
At least one analgesic (PP) (%)	52.0	58.0	48.7	36.0	9.3 (±11.0) n.s.	22.0 (±13.4) **	12.7 (±15.0) n.s.
At least one analgesic (PRN) (%)	68.8	72.3	64.3	64.1	−8.0 (±10.4) n.s.	−8.2 (±13.1) n.s.	−0.2 % (14.7) n.s.

**p < 0.01, *p < 0.05, *n.s.* not significant, *Diff* Group difference, *FR* Fluctuation range of 95 % confidence interval

^a^ cognitively unimpaired/slightly impaired, ^b^ cognitively moderately to severely impaired, verbally communicating, ^c^ cognitively moderately to severely impaired, not verbally communicating

The most frequently prescribed analgesics (PP and/or PRN) were weak cyclooxygenase (COX) inhibitors (Metamizol, Paracetamol), found in 80.1 % of residents with at least one analgesic prescription, where the differences between the cognition-groups were negligible. Topical analgesics (e.g. diclofenac gel) were used by 40.5 % of all residents with analgesic medication and 39.9 % used opioids. Systemically administered NSAIDs were prescribed in 36.7 % of all residents with at least one analgesic prescription. Use of NSAIDs differed significantly between all groups, with 44.4 % in group CUS, 28.9 % in group CI-V and 18.4 % in group CI-NV. Other pharmacological painkillers were rarely used and due to small number not further statistically analyzed (Table 5).

Focusing on PP analgesics, the most frequently prescribed subgroups were opioids, found in 50.2 % of all residents with PP analgesics, followed by topical analgesics with 47.5 %, weak COX-inhibitors with 32.3 % and NSAIDs with 26.7 %. There were no significant differences between the cognition-groups.

Looking at the PRN analgesics, the most common subgroup were weak COX-inhibitors prescribed for 82.6 % without differences between the cognition-groups. Opioids as PRN were prescribed to 14.6 % of group CI-NV, in contrast to 20.9 – 27.2 % for CUS and CI-V, respectively. The only significant group difference in PRN analgesics was between group CUS (35.5 %) and group CI-V (20.3 %) regarding NSAIDs (Table 5).

**Table 5:** Subgroups of analgesics - descriptive statistics

Subgroup of analgesics^a^					
		all	CUS^b^	CI-V^c^	CI-NV^d^
weak cyclooxygenase (COX) inhibitors (Metamizol, Paracetamol)	PP and PRN	80.1 %	79.7 %	78.9 %	83.7 %
	PP	32.3 %	31.2 %	35.7 %	30.4 %
	PRN	82.6 %	81.4 %	81.1 %	90.2 %
Topical analgesics	PP and PRN	40.5 %	43.0 %	38.9 %	32.7 %
	PP	47.5 %	49.3 %	48.2 %	34.8 %
	PRN	15.7 %	15.7 %	23.2 %	22.0 %
Opioids	PP and PRN	39.9 %	41.1 %	42.2 %	30.6 %
	PP	50.2 %	50.0 %	50.0 %	52.2 %
	PRN	21.6 %	20.9 %	27.2 %	14.6 %
NSAIDs	PP and PRN	36.7 %	44.4 %*	28.9 %*	18.4 %*
	PP	26.7 %	30.4 %	23.2 %	13.0 %
	PRN	29.3 %	35.5 %*	20.3 %*	19.5 %

^a^Percentages are based on residents who had at least one prescription of each subgroup (PP and PRN, PP, PRN); significant test results are marked with *(*p* < 0.05)

^b^ cognitively unimpaired/slightly impaired, ^c^ cognitively moderately to severely impaired, verbally communicating, ^d^ cognitively moderately to severely impaired, not verbally communicating

**Fig. 2:**
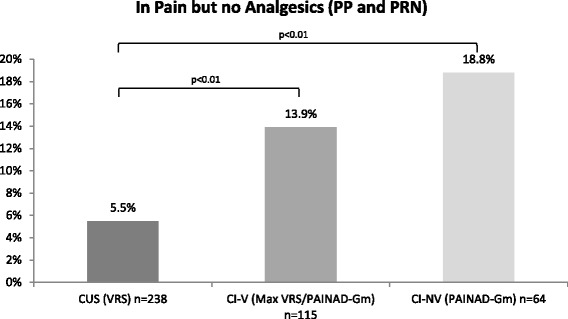
Residents presenting pain without any medical pain treatment in relation to cognitive groups. Notes: overall sample had 8 missing data

The rate of residents without any analgesic medication PP or PRN despite presenting pain differed between the cognition-groups, increasing from 5.5 % in group CUS to 13.9 % in group CI-V and 18.8 % in group CI-NV (Fig. 2). The probability of having no analgesic prescription despite indicated pain was significantly higher in both groups of CI residents than in CUS residents and it differed with regard to the ability to verbalize pain: residents of group CI-V were at a 2.6-fold higher risk, residents of group CI-NV at a 3.4-fold higher risk to be affected (Table 6).

**Table 6:** No analgesics though in pain - Associations between cognition groups *(Log-binomial regression)*

No analgesics despite pain				
	Wald-Chi^2^	*p*-value	RR	(95 % CI)
CI-V^b^ in relation to CUS^a^	6.9	**<0.01**	2.6	(1.3 – 5.1)
CI-NV^c^ in relation to CUS^a^	10.8	**<0.01**	3.4	(1.6 – 7.2)

*RR* Risk Ratio, *CI* Confidence Interval

^a^ cognitively unimpaired/slightly impaired, ^b^ cognitively moderately to severely impaired, verbally communicating, ^c^ cognitively moderately to severely impaired, not verbally communicating

### Antipsychotics

Notably, antipsychotics were commonly used in the residents of the investigated NHs. 37.0 % of CUS residents were prescribed at least one antipsychotic PP with 47.0 and 51.6 % as the corresponding numbers for groups CI-V and CI-NV, respectively. Although the probability for CI residents to receive antipsychotics was similar to that of CUS residents, the test results indicate a trend (*p* < 0.1) toward more prescriptions for CI-V residents and a significant increase (*p* < 0.05) in risk of antipsychotic prescription for CI-NV residents (Table 7).

**Table 7:** Antipsychotics PP - Associations between cognition groups *(Log-binomial regression)*

Antipsychotics PP				
	Wald-Chi^2^	*p*-value	RR	(95 % CI)
CI-V^b^ in relation to CUS^a^	3.4	**<0.1**	1.3	(1.0 – 1.6)
CI-NV^c^ in relation to CUS^a^	5.1	**<0.05**	1.4	(1.0 – 1.9)

*RR* Risk Ratio, *CI* Confidence Interval

^a^ cognitively unimpaired/slightly impaired, ^b^ cognitively moderately to severely impaired, verbally communicating, ^c^ cognitively moderately to severely impaired, not verbally communicating

## Discussion

Our data confirm that pain is still a frequent symptom in Austrian NH residents. More than two thirds either self-report pain (VRS) or indicate prevalence of pain on the PAINAID-Gm observational assessment. These findings are in line with previously published data [37, 40].

In addition, reports of residents on their pain varied depending on cognitive function and pain-assessment instruments. Assessing moderately to severely cognitively impaired individuals’ pain under standardized conditions resulted in a high frequency of pain (approximately 80 %) compared to the literature [5, 6, 41]. Since CI-V residents’ observational assessment showed significantly higher pain prevalence than their self-report, the true ratio of CI-V residents in pain is at issue. Against this background and considering communication deficiencies in this group, the possibility of higher pain prevalence than indicated through self-report should be considered. By assuming the presence of pain, if at least one of both instruments indicates pain – as was done in this study – sensitivity is aimed for, however, specificity of pain detection may be lowered.

In contrast to literature [12], our results demonstrate differences in pain-associated diagnoses relating to cognitive state with significantly more diagnoses indicating pain in the CUS group in comparison to the CI-V group. This discrepancy cannot be interpreted from our data, but may reflect a decreasing awareness in determining medical diagnoses for the cognition-groups with advanced decline.

Our data adds to the evidence that persons with advanced cognitive decline less often receive pain treatment [12, 15–19] and, is therefore, conflicting with reports from other European countries suggesting that analgesic use is higher among people with cognitive impairment as compared to older adults without cognitive impairment [8, 20, 42]. This may be explained by differences in national prescription habits and configuration of medical supply. It has to be pointed out though that frequency of analgesic prescription is not necessarily a sign, whether (or not) an adequate analgesic treatment is being prescribed to the right people at the right time [7].

It is noteworthy to add that we investigated the percentages of permanently scheduled analgesics (PP) and analgesics prescribed ‘as needed’ (PRN) in relation to the different cognition-groups. In all groups, more analgesics were prescribed PRN (64.1 – 72.3 %) than PP (35.9 – 58.0 %), with no significant group differences for the PRN prescriptions. In contrast, PP prescriptions dropped with cognitive decline, with 58.0 % for group CUS, 48.7 % for group CI-V and 36.0% for group CI-NV. The difference between groups CUS and CI-NV reached significance suggesting that advanced stages of cognitive decline are associated with a reserved attitude to medical treatment. This is also supported by the mean numbers of prescriptions, which exhibit a decline from group CUS (9.7 ± 3.9) to groups CI-V (8.8 ± 3.9) and CI-NV (6.8 ± 3.2), again reaching significance between CUS and CI-NV.

According to the literature, pain medication in individuals with cognitive impairment is generally of low dosage and stronger drugs such as opioids are less likely to be considered [7]. In our sample, the most frequently prescribed PP pharmacological subgroups were opioids with 50.2 %, followed by topical analgesics with 47.5 %, weak cyclooxygenase inhibitors (Metamizol, Paracetamol) with 32.3 % and NSAIDs with 26.7 %. No differences between the cognition-groups were detectable. Notably, the number of opioid prescriptions is remarkably high in comparison to recent literature [15, 19], as is the overall number of PP in our study. The high number of opioid prescriptions is in accordance with other investigations reporting an increasing opioid use in several countries [43].

Merging PP and PRN prescriptions, the use of NSAIDs diminishes significantly with the loss of cognitive function, showing a prevalence of 44.4 % in group CUS compared to 28.9 and 18.4 % in groups CI-V and CI-NV, respectively. This trend also partially applies to PRN prescriptions. The lower NSAID prescription behavior as cognition declines could, however, be also due to the recommendation to prescribe this class of medication less frequently due to its detrimental side-effects on particularly the geriatric population [44–47].

Cognitively impaired individuals seem to experience intensity and affective components of pain differently than their cognitively fit counterparts [7]. Moreover, the decline in the ability to communicate results in considerable challenges for detecting pain, particularly in advanced stages of cognitive impairment. Cognitively impaired individuals may embody pain by BPSD, such as agitation, denial or withdrawal, giving rise to misinterpreting pain as a psychiatric condition and resulting in the inappropriate treatment with antipsychotic medication [7, 21].

In our sample, prescription of antipsychotics was strikingly common, as reported previously for Austrian NH residents by Richter (2012) [48]. The high number of antipsychotic prescriptions is likely to be an indicator for a perceived or actual lack of strategies to manage BPSD [48]. Antipsychotics were prescribed in nearly half of the participants of our study with the difference between CI-V (47.0 %) and CUS (37.0 %) residents demonstrating a trend and the difference between CI-NV (51.6 %) and CUS (37.0 %) residents reaching statistical significance. In addition, residents with advanced cognitive decline had a significantly higher risk of suffering from pain without having an analgesic prescription, giving rise to the assumption that pain-associated BPSD indicate antipsychotic rather than analgesic treatment. This finding adds to an ongoing discussion, since some studies demonstrated that treatment of pain might decrease incidence and severity of BPSD [22, 49], whereas a recently published meta-analysis does not support strong associations between pain and BPSD [50].

To our best knowledge, our investigation is the first of its kind to address the relationship between prevalence of pain, prescription of analgesics and the application of different assessment tools adjusted to the cognitive state of NH residents. Connecting these aspects, CI-V residents had a 2.6-fold higher risk of suffering from pain without having any analgesics prescribed as compared to CUS residents; for CI-NV residents, this risk was even 3.4-fold. These findings suggest cognitive impairment as a hindering factor for receiving sufficient medical pain treatment. Against the background of communication deficiencies, these considerable disparities may be traced back to lacking or inadequate pain detection, which further points toward the necessity of meticulous and standardized pain assessment adapted to the residents’ cognitive state as a prerequisite of adequate treatment. Moreover, such an assessment is mandatory to find the balance between sufficient analgesia, side effects and futile treatment, since patients are often restricted in reporting the effect of therapy [40]. Along these lines, recording the effect of pain treatment over time might be a pivotal measure to warrant optimized pain management and to avoid inadequate medications.

We concede some limitations to our work: Due to the cross-sectional study design, it is difficult to make causal conclusions about the data. Additionally, considering nursing homes in Austria, it should be noted that requirements related to staffing and facility structure are regulated by federal law. Although our sample only consisted of nursing homes from one Austrian nursing home operator, which limits its generalizability, differences in staffing and facility structure between the nursing homes in our sample and other nursing homes are expected to be rather negligible. The quality of the medical documentation in the nursing homes did not allow the use of standardized tools to rate cumulative illness, thus, limiting the comparability of disease burden between the cognition- groups. Moreover, the medical documentation only included information about prescribed medication, but not if the prescribed medication was actually given to or taken by the resident. The classification of cognitive abilities was based on the current assessment of the MMSE. Therefore, definitive assignment to clinical entities or etiology is not warranted. The use of antipsychotic drugs might be biased by the presence of BPSD, which were not recorded systematically in the medical documentation of the nursing homes. Due to different cognitive states of the participants different pain assessment methods were used.

## Conclusions

Results point toward an underuse of pain medication in nursing home residents with advanced cognitive decline, especially those unable to communicate pain verbally. The implementation of standardized pain assessments adapted to the cognitive abilities of residents may foster the recognition of pain, warrant optimized pain management, reduce inadequate medication and consequently raise the chance of equally effective pain treatment regardless of cognitive state.

## Abbreviations

ATC, anatomical therapeutic chemical; BPSD, behavioral and psychological symptoms in dementia; CAPI, computer assisted personal interview; CI, cognitively moderately – severely impaired; CI-NV, cognitively moderately – severely impaired/not verbally communicating; CI-V, cognitively moderately – severely impaired/verbally communicating; COX, cyclooxygenase; CUS, cognitively unimpaired, slightly impaired; MMSE, mini mental status examination; n, sample size; NH, nursing homes; NSAID, nonsteroidal anti-inflammatory drugs; OSiA, optimiertes schmerzmanagement in altenpflegeheimen; PAINAD-Gm, pain assessment in advanced dementia (modified German version); PP, prescriptions scheduled permanently; PRN, prescribed ‘as needed’; RR, risk ratio; VRS, verbal rating scale (pain assessment); WHO, World Health Organization
